# Single-Anastomosis Sleeve Jejunal (SAS-J) Bypass as Revisional Surgery After Primary Restrictive Bariatric Procedures

**DOI:** 10.1007/s11695-022-06123-8

**Published:** 2022-06-06

**Authors:** Alaa M. Sewefy, Ahmed M. Atyia, Taha H.Kayed, Hosam M. Hamza

**Affiliations:** Department of Surgery, Minia University Hospital, Minia, postal code: 61511 Egypt

**Keywords:** Revisional bariatric surgery, Single-anastomosis sleeve jejunal bypass, SASI, Sleeve loop bipartition

## Abstract

**Purpose:**

Single-anastomosis sleeve jejunal (SAS-J) bypass is the modification of a single-anastomosis sleeve ileal (SASI) bypass with a short biliary limb. SAS-J bypass is reported to be a good primary bariatric procedure. This study aimed to evaluate the results of SAS-J bypass as a revisional surgery after failed primary restrictive bariatric procedures.

**Material and Methods:**

This was a prospective cohort study including 43 patients who underwent SAS-J bypass as a revisional surgery for weight regain after laparoscopic sleeve gastrectomy (LSG), laparoscopic adjustable gastric band (LAGB), or laparoscopic gastric plication.

**Results:**

Of the total patients, 35 (81.4%) were female, and 8 (18.6%) were male. The mean BMI was 46.3 kg/m^2^. The mean age was 41 years. Thirty-two patients (74.4%) had a failed sleeve, 9 (20.9%) had a failed LAGB, and 2 (4.7%) had a failed gastric plication. The mean operative time was 104 min. Intra-abdominal bleeding occurred in 1 case (2.3%), and intraluminal bleeding occurred in 3 cases (7%). No case (0%) developed a leak. The percentage of excess weight loss (%EWL) reached 76.5% after 1 year. Type 2 diabetes mellitus remission occurred in all diabetic patients, hypertension remitted in 80%, hyperlipidemia remitted in 83.3%, and obstructive sleep apnea syndrome improved in all cases. Gastroesophageal reflux disease (GERD) symptoms were improved in 86.7% of patients. Significant biliary gastritis occurred in 4 patients (9.3%). Dumping syndrome was reported in 4 patients (9.3%).

**Conclusions:**

SAS-J bypass was effective as a salvage surgery after failed restrictive bariatric procedures, but long-term follow-up is needed.

**Graphical abstract:**

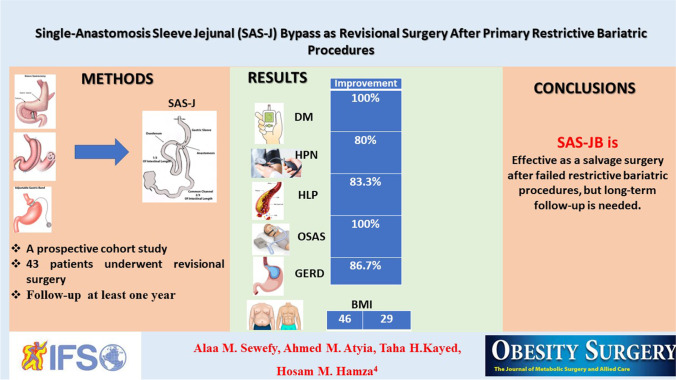

## Introduction

Obesity is a worldwide health problem, usually associated with serious comorbidities such as type 2 diabetes mellitus (T2DM), hypertension, obstructive sleep apnea syndrome (OSAS), hyperlipidemia, and osteoarthritis. Surgical intervention is the most effective long-term treatment for morbid obesity and its comorbidities [1].

Bariatric surgeries are either purely restrictive, purely malabsorptive, or combined. Restrictive procedures are generally considered safe, quick, and easy to learn, but they are associated with a high rate of weight regain [2].

In the last decade, the number of bariatric operations has increased dramatically. This increase is associated with the high reoperation rate due to complications, failure of weight loss, or weight regain, especially after primary restrictive procedures, such as vertical banded gastroplasty (VBG), laparoscopic adjustable gastric banding (LAGB), or laparoscopic sleeve gastrectomy (LSG) [3].

The definition of weight regain has not yet been established in the literature, but around one-third of studies define it as a BMI of ≥ 35 kg/m^2^ or EWL of ≤ 50% [4, 5]. The reported failure rates after LSG, VBG, and LAGB are up to 30–60% [6–12]. Revisional procedures have moderate efficacy for weight loss, with a higher complication rate and longer length of stay compared to primary bariatric interventions [4, 13, 14]. Duodenal switch (DS), Roux-en Y gastric bypass (RYGB), one-anastomosis gastric bypass (OAGB), and single-anastomosis duodeno-ileal (SADI) bypass are the most common bariatric procedures for revisional surgery. SAS-J bypass is effective as a primary bariatric procedure, with many advantages, including its relative simplicity, less malnutrition compared to other malabsorptive procedures, and easy screening of the upper GIT and biliary tree [15]. This study aimed to evaluate SAS-J bypass as a revisional procedure.

## Materials and Methods

This was a prospective cohort study of 43 patients who underwent SAS-J bypass as a revisional procedure after weight regain or failure of LSG, gastric plication, or LAGB between January 2018 and January 2021. Of the total number, 5 cases were operated in our center, and the other 38 were operated in other centers. Failure was considered when EWL was < 50%, BMI remained ≥ 35 kg/m^2^, or control of obesity-related comorbidities was not satisfactory. Weight regain was defined as an increase in BMI to > 35 kg/m^2^ after successful weight loss [4, 5, 16]. In this study, all patients asked for revisional surgery, mainly for weight regain after initial satisfactory weight loss ± comorbidities (as shown in Table 1). Weight regain was due to patient noncompliance with lifestyle change and follow-up. All patients had a psychological consultation. They were informed that lifestyle change is the mainstay of long-term results and consented to this. The primary operations were done at least 5 years before the revisional operation. All patients had at least one trial of a weight-loss diet for 3–6 months with a nutritionist. The study received approval from our institutional review board, and all patients gave informed written consent. All procedures performed in this study were in accordance with the ethical standards of the institutional and national research committee and with the 1964 Helsinki declaration and its later amendments or comparable ethical standards. Routine CT gastric volumetry was done in all cases. Upper GIT endoscopy was done in all gastric band cases and symptomatic LSG cases. All cases were operated by the same surgeon.

**Table 1:** Indications for the revisional surgery

Indication for surgery	1ry procedure		
	LSG (No. = 32)	LAGB (No. = 9)	Gastric plication (No. = 2)
Weight regain	32	9	2
+ DM	3	1	1
+ HPN	4	1	0
+ SAS	2	0	1
+ GERD	9	6	0
+ Hyperlipidemia	11	6	1

*DM* diabetes mellitus, *HPN* hypertension, *SAS* sleep apnea syndrome, *GERD* gastroesophageal reflux disease

### Surgical Technique

The patient was placed in the French position with a steep reverse-Trendelenburg position. The surgeon stood between the patient’s legs. All patients were operated on under general anesthesia with endotracheal intubation. The camera port was inserted cautiously using an optical trocar, and the other working ports were entered under direct vision.

#### SAS-J Bypass After LAGB

Dissection of adhesions was done cautiously. If the adjustable band was present, it was removed first, together with its capsule or at least the anterior side of the capsule. After complete dissection of all adhesions to free the stomach from the liver anteriorly, we began with the division of the greater omentum from the stomach and continued division upward to the left crus of the diaphragm. The crus was completely cleared from any adhesions. The stomach was completely freed posteriorly by dissecting any adhesions between it and the pancreas. The dissection continued downward toward the pylorus. A 36 French calibration tube was used for the proper sleeve. The stapling was initiated 6 cm from the pyloric ring and continued using suitable reload colors according to the stomach thickness. Stapling was continued until it completely divided the stomach. We routinely over-sewed the staple line using a running 3–0 Prolene suture. The sleeved stomach was routinely fixed to the crus of the diaphragm, to prevent later migration of the stomach into the chest and decrease the incidence of reflux, and also fixed to the peripancreatic fascia to prevent its twisting. Division of ligaments that fix the stomach during LSG may lead to gastric torsion and postoperative emptying disorders. Gastric volvulus has been already reported after LSG [17, 18].

**Fig. 1:**
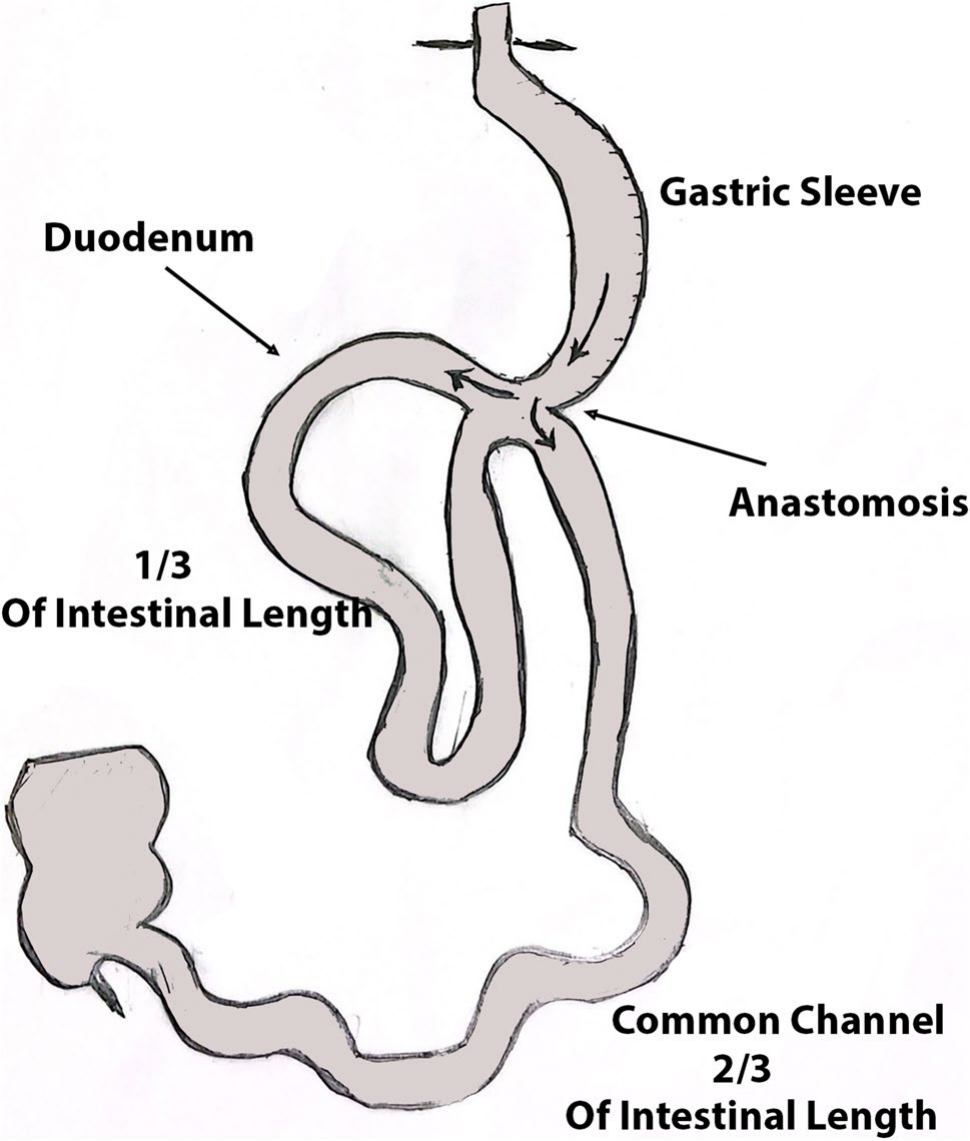
Schematic demonstration of SAS-J bypass

The duodenojejunal (DJ) junction was then identified, and we measured the total intestinal length from the DJ by the number of counts instead of using a measure. We then took nearly one third of the total count from the DJ junction (for example, if the total intestinal count was 100, the anastomosis was done at 33 counts from the DJ). This process standardized the procedure to a percentage rather than a fixed length (see Fig. 1), since the common limb may be short in some and longer in others. Some patients have a much smaller or much longer total bowel length. The jejunum was fixed at the desired length with an orientation stitch to the pylorus. The anastomosis between the jejunum and sleeved stomach was performed at the dissected inferior border of the sleeved stomach, using a 45-blue reload at around 1–2 cm from the pyloric ring, to make an approximately 40-mm stoma. Early in our practice of SAS-J, we noticed unsatisfactory weight loss using 30 reload. The defect in the gastro-jejunal anastomosis was then closed with a two-layer running suture. Finally, another orientation stitch was made at the left side of the anastomosis between the jejunum and the staple line of the stomach. The aim of these two orientation stitches was as follows: (1) to minimize tension on the anastomosis, (2) to make the anastomosis and the jejunal loop anatomically oriented without twisting, and (3) as an anti-reflux measure by the left stitch. Finally, a methylene blue leak test was performed.

#### SAS-J Bypass After LSG and Plication

In cases of previous sleeve, the first step after the dissection of adhesions was to completely free the stomach. We evaluated the size of the stomach; if it was not hugely dilated, we plicated it on a 36 French calibration tube ± fundectomy and/or antrectomy. If the stomach was hugely dilated, we resleeved it using a suitable reload according to the thickness of the stomach. The stomach was evaluated by preoperative gastric volumetry and also intraoperatively by insertion of a 36 French calibration tube. If enough stomach was redundant for stapling, resleeve was performed; if not, we did either overall plication or fundectomy and/or antrectomy + body plication.

In cases of previous gastric plication, the plication was first untied, then we continued sleeve gastrectomy and sleeve jejunal anastomosis as described.

Early ambulation and clear fluids were initiated 5 h after surgery. PPIs were administrated for 3 months postoperatively.

### Data Collection

Data collected included age, sex, body weight and height, obesity-related comorbidities, presence of gallstones, operative time, conversion, intraoperative and postoperative complications, and postoperative data including weight loss and improvement of comorbidities.

### Study Outcomes

The primary outcome studied was the percentage of excess weight loss (%EWL). Secondary outcomes included operative time, perioperative complications, remission of obesity-related comorbidities, and late complications.

#### Definition of Outcomes

Weight loss was reported as %EWL and total weight loss percentage (%TWL). The %EWL was calculated as [(preoperative weight − follow-up weight) / preoperative weight − ideal weight] × 100. The %TWL was calculated as [(preoperative weight − follow-up weight) / preoperative weight] × 100 [15].

Hypertension remission was defined as systolic blood pressure of ≤ 130 mmHg or diastolic blood pressure of ≤ 85 mmHg without the use of any medications. T2DM remission was defined as a fasting glucose level < 5.6 mmol/L and a glycosylated hemoglobin value < 6.5% without the use of any medications. T2DM improvement was defined as a decrease in the number or dosage of medications. DSL remission was defined as fasting total cholesterol, low-density lipoprotein, and triglyceride levels of < 200 mg/dL, < 150 mg/dL, and < 170 mg/dL, respectively, and high-density lipoprotein levels of > 30 mg/dL. OSAS resolution was defined as the cessation of continuous positive airway pressure mask use due to the normalization of OSAS, assessed through ventilation analysis by a pneumologist.

GERD was determined based on symptoms including regurgitation, retrosternal chest pain, and complications such as esophagitis seen on endoscopy; GERD remission was defined as the remission of clinical symptoms with cessation of medications. The Arabic version of the GERDQ questionnaire was used preoperatively and 3 months postoperatively to evaluate the degree of GERD [16, 19]. The presence of dumping syndrome was determined when a score of > 7 was obtained on Sigstad’s scoring system [20].

### Follow-up

All patients were followed up in the clinic weekly for 1 month, then monthly in the first year. In the second year, follow-up was every 3 months. After the second year, the follow-up was every 6 months, either in the clinic or by telephone call or WhatsApp message. If any patient developed symptoms between their follow-ups, they were also seen in the clinic. The minimum follow-up period was 1 year. All patients were allowed a liquid diet for 2 weeks, then a soft diet for the following week. Subsequently, patients took a high-protein, low-calorie diet. Additional food was gradually added under dietitian supervision. High-concentration multivitamin supplementations were prescribed to be taken regularly.

All patients had follow-up investigations every 3 months, including a complete blood picture, liver function, serum vitamin D, lipid profile, serum albumin, fasting blood sugar, HBA1c, serum calcium, and serum iron. Any additional investigations were ordered according to the patient’s clinical condition. We also recorded any early or delayed complications.

### Statistical Analysis

The data were analyzed using IBM SPSS (version 24) for Windows. Data were expressed as percentages or mean ± standard deviation. Suitable statistical analysis methods were used for parametric and non-parametric procedures, with Student’s *t*-test used to compare continuous variables and a chi-square test used to compare categorical or ordinal variables. *p* values of < 0.05 were considered statistically significant.

## Results

### Preoperative Data

This study included 43 patients who underwent SAS-J bypass as a revisional procedure after failed LAGB, LSG, or gastric plication between January 2018 and January 2021. Of the patients, 32 (74.4%) had a failed sleeve, 9 (20.9%) had a failed LAGB, and 2 (4.7%) had a failed gastric plication. In all cases, weight regain was the main cause of revision (Table 1). Thirty-five (81.4%) patients were female, and 8 (18.6%) were male. The mean BMI was 46.3 kg/m^2^, and the mean age was 41 years. T2DM was present in 5 patients (11.6%), hypertension in 5 (11.6%), gallstones in 2 (4.7%), OSAS in 3 (7%), GERD in 15 (35%), and hyperlipidemia in 18 (42%; see Table 2).

**Table 2:** Preoperative characteristics of all patients

Variables		Value (total number = 43)
Age		41 ± 6
Sex	F	35 (81.4%)
	M	8 (18.6%)
Primary procedure	LSG	32 (74.4%)
	LAGB	9 (20.9%)
	Gastric plication	2 (4.7%)
Weight		126 ± 13
Height in meter		1.66 ± 0.06
BMI		46 ± 3
Comorbidities	Diabetes	5 (11.6%)
	Hypertension	5 (11.6%)
	GERD	15 (35%)
	Hyperlipidemia	18 (42%)
	Sleep apnea	3 (7%)
Gallstone		2 (4.7%)

*F* female, *M* male

### Operative and Early Postoperative Results

Preoperative gallstones were present in 2 (4.7%) patients, who underwent cholecystectomy in the same session with no complications. The mean operative time was 104 min, with almost all cases discharged the next day. Intra-abdominal bleeding occurred in 1 case (2.3%); laparoscopic exploration revealed only a large peri-gastric hematoma, and evacuation was performed without locating an apparent source of the bleeding. Intraluminal bleeding occurred in 3 cases (7%); these patients presented with rectal bleeding on the second postoperative day. The bleeding was managed by conservative treatment. No case (0%) developed a leak (Table 3).

**Table 3:** Intraoperative variables and complications

Variables		Value (total number = 43)	
Associated lap chole		2 (4.7%)	
Operative time		104 ± 23	
Return to work		10 ± 2 days	
Complications		Incidence	Grade
Early	Leakage	0 (0%)	III
	Intra-abdominal bleeding	1 (2.3%)	III
	Intramural bleeding	3 (7%)	III
Late	Biliary gastritis	4 (9.3%)	I
	Dumping	4 (9.3%)	I
	Iron deficiency	3 (7%)	I
Total	15 (34.8%)		

NB: Complication grading is according to Clavien-Dindo

### Short-term Effect on BMI and Comorbidities

**Fig. 2:**
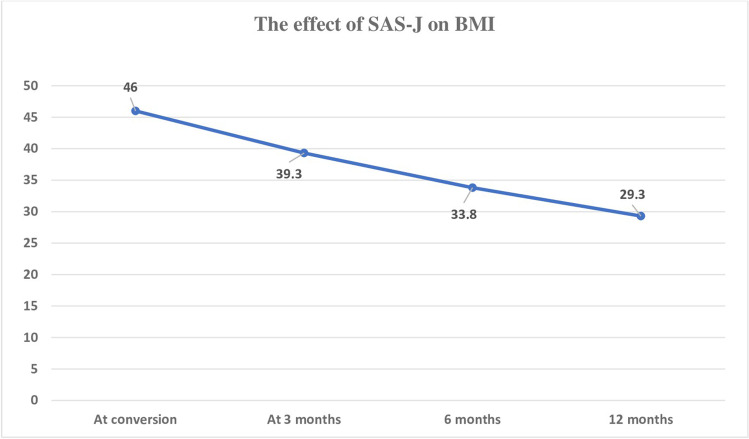
The effect of SAS-J bypass on BMI

The mean %TWL was 30% and the mean %EWL reached 76.5% for all included patients after 1 year, the minimum follow-up period (Fig. 2). In 27 patients who completed 2 years of follow-up, the %EWL reached 77.6%, and the %TWL was 32.7%. T2DM remission occurred in all diabetic patients within 3 months of surgery, hypertension remitted in 80%, hyperlipidemia remitted in 83.3%, and OSAS improved in all cases at 1 year of follow-up. GERD symptoms were improved in 86.7% of patients (Tables 4 and 5).

**Table 4:** The effect of SAS-J bypass on weight loss

	At time of conversion No. = 43	3 months postoperative No. = 43	6 months postoperative No. = 43	12 months postoperative No. = 43
BMI	46 ± 3	39.3 ± 1.5	33.8 ± 1	29.3 ± 2
%TWL	-	9 ± 1	20.5 ± 3	30 ± 5.8
%EWL	-	17.2 ± 4	55.3 ± 3	76.5% ± 9%

Each *p* value was calculated by paired *t*-test. We compared each value with just before follow-up values

**Table 5:** The effect of SAS-J bypass on comorbidities

	Preoperative No. = 43	At 3 months No. = 43	6 months No. = 43	12 months No. = 43	% of improvemen**t**	*p*value
Diabetes	5/43 (11.6%)	0	0	0	100%	< 0.02
Hypertension	5/43 (11.6%)	1	1	1	80%	< 0.09
Hyperlipidemia	18 /43(42%)	16	6	3	83.3%	< 0.001
Sleep apnea	3/43 (7%)	2	0	0	100%	< 0.07
GERD	15/43(35%)	2	2	2	86.7%	< 0.001

*p* value is significant when ˂ 0.05%. Each *p* value was calculated by chi-square test. We compared each the preoperative incidence with the incidence at 1-year follow-up

### Late Complications

Sporadic significant biliary gastritis occurred in 4 patients (9.3%), with biliary vomiting and epigastric pain. All responded to conservative treatment in the form of a light liquid diet, a PPI (40 mg/day), sucralfate (1 g before every meal and before bedtime), and antiemetics. Patients usually improved within 2 weeks and gradually returned to a normal, healthy diet. Dumping syndrome was reported in 4 patients (9.3%) and improved with time. Iron deficiency anemia was reported in 3 patients (7%) and improved with supplementation (Table 3).

## Discussion

Weight loss failure and weight regain may occur in the long term following restrictive procedures. These restrictive procedures were converted due to either weight loss failure or surgical complications [4, 21].

DS, RYGB, OAGB, and SADI are the commonest reported bariatric procedures for revisional surgery. In our original study, SAS-J bypass was very effective as a primary bariatric procedure, with many advantages, including its relative simplicity, less malnutrition compared to other malabsorptive procedures, and easy screening of the upper GIT and biliary tree [15]. This is the first study to discuss the use of SAS-J bypass as a revisional procedure after failed restrictive operations.

At 1 year of follow-up, the mean %TWL was 30% and the mean %EWL was approximately 76.5%, compared to the reported %EWL of 85% with SAS-J bypass as the primary procedure in our original study, 64–79% after SADI-S, and 61–66% after RYGB [5, 15, 22–27]

Regarding the effects of conversion on T2DM, the remission rate in this study was 100%, compared to 85–100% after revisional OAGB, 60% after revisional RYGB, 88% after SADI, and 100% in SAS-J as the primary procedure [5, 15, 22–27].

Improvements have been observed in other comorbidities, such as hypertension, with remission rates of 58–94% at 5 years following the revisional surgery [4]. Hypertension remission in this study was 80% compared to 40–100% in revisional OAGB and 89% in primary SAS-J bypass [15, 25–27].

The rate of lipid profile improvement in this study was 83.3%, compared to 25–61.5% after revisional RYGB and OAGB. In another study, the remission rate of dyslipidemia after revision of failed LSG or LAGB to OAGB was 56%, compared to 100% in primary SAS-J bypass [4, 15, 26].

Improvement of OSAS occurred in all patients, compared to average remission rates of 66–80% after revisional OAGB, 73.7% after revisional RYGB, and 100% after primary SAS-J bypass [1, 15, 26, 27].

GERD improved in 87.7% of patients in this study, compared to 82% in revisional OAGB, up to 75–100% in RYGB, and 86.7% in SAS-J bypass as the primary procedure [4, 15, 28, 29].

The perioperative complication rates after revisional bariatric surgery (RBS) are significantly higher than after primary bariatric surgery. In this study, the early complication rate was 9.3%, compared to 29.5% after revisional RYGB and 10.5% after revisional SG in a systematic review conducted by Mahawar et al. [30]. A study by Zhang et al. compared revisional and primary RYGB and found that the postoperative complication rates were 55% vs. 28%, readmissions were 16% vs. 7%, and reoperations were 16.9% vs. 3.2% [31].

Anastomotic or staple-line leaks are the most feared complication following RBS. In this study, the rate was 0%, compared to 5.8% after revisional RYGB [31]. This may be due to oversewing of the staple line or the small sample size.

Transient biliary gastritis is the most common delayed complication associated with revisional SAS-J bypass, at 9.3%, compared to 7% in primary SAS-J bypass [15]. The rate of biliary gastritis after OAGB ranges from 0.9 to 30% [32, 33].

In this study, the incidence of iron deficiency was 7%, compared to 18–53% after RYGB, around 23.7% after OAGB, and 1–54% after sleeve gastrectomy [34, 35].

## Limitations

The study design would have been better as a clinical trial comparing the results of SAS-J bypass with other revisional procedures. The sample size was small, so the study may not reflect the actual results of the technique. The follow-up period was short, and the technique needs longer-term follow-up to show effective results.

## Conclusion

SAS-J bypass is effective as a salvage surgery after failed restrictive bariatric procedures, but long-term follow-up is needed.
